# Optical Properties of Diatom Nanostructured Biosilica in *Arachnoidiscus* sp: Micro-Optics from Mother Nature

**DOI:** 10.1371/journal.pone.0103750

**Published:** 2014-07-30

**Authors:** Maria Antonietta Ferrara, Principia Dardano, Luca De Stefano, Ilaria Rea, Giuseppe Coppola, Ivo Rendina, Roberta Congestri, Alessandra Antonucci, Mario De Stefano, Edoardo De Tommasi

**Affiliations:** 1 Institute for Microelectronic and Microsystems, Department of Naples, National Research Council, Naples, Italy; 2 Laboratory of Biology of Algae, Department of Biology, University of Rome “Tor Vergata”, Rome, Italy; 3 Department of Environmental, biological, and Pharmaceutical Sciences and Technologies, Second University of Naples, Caserta, Italy; Universita’ degli Studi del Salento, Italy

## Abstract

Some natural structures show three-dimensional morphologies on the micro- and nano- scale, characterized by levels of symmetry and complexity well far beyond those fabricated by best technologies available. This is the case of diatoms, unicellular microalgae, whose protoplasm is enclosed in a nanoporous microshell, made of hydrogenated amorphous silica, called frustule. We have studied the optical properties of *Arachnoidiscus* sp. single valves both in visible and ultraviolet range. We found photonic effects due to diffraction by ordered pattern of pores and slits, accordingly to an elaborated theoretical model. For the first time, we experimentally revealed spatial separation of focused light in different spots, which could be the basis of a micro-bio-spectrometer. Characterization of such intricate structures can be of great inspiration for photonic devices of next generation.

## Introduction

Until few years ago, study and characterization of micro-and nano-structures found in living organisms belonged to taxonomists, i.e. scientists devoted to their classification: results were always interpreted in terms of peculiar characters for species identification and evolution [1], [2]. Nowadays, more and more research groups focus their investigations on nanostructured constituents of animals or plants: in many instances, nature offers elegant and unique solutions that by far exceed modern technological design. Just to cite consolidate examples, lotus leaf effect [3], biomineralization processes [4], and topology optimization [5] are only few among hundreds of bio-inspiring phenomena recently published in literature. Beyond bio-mimicry [6], on the other hand, rare attempts have been done to directly use components of living organisms as technological devices: is this the case of butterfly scales and diatoms microshells. Portyrailo and coauthors demonstrated highly selective vapour response and infrared imaging by using Morpho butterfly wings as optical reflectors [7], [8]; Rorrer’s team used antibody functionalized diatom microshells to detect biomolecules [9]; our group succeeded in monitoring gas in surrounding environment with very high sensitivity by diatom photoluminescence [10]–[12]. More than butterflies (about 20.000 species existing), diatom-based applications could benefit of a huge variety of morphologies and shapes, due to over 200.000 classified species distributed in all aquatic systems as in some subaerial habitats. Diatoms are unicellular microalgae, whose protoplasm is enclosed in a microshell, called frustule, made of amorphous hydrated nanoporous silica [2]. A frustule is constituted by two halves (*valves*), connected by a lateral girdle. Both valves and girdles show specie-specific, hierarchical, complex and quasi-ordered patterns of pores (*areolae*) whose dimensions can range from nanometer to micrometer scale. Diatoms can be distinguished on the basis of frustule shape and simmetry of pore patterns: centric are all diatoms whose frustule has radial symmetry, while pennate are bilaterally symmetric. While frustule formation mechanisms have been deeply studied [13]–[15], its role and evolutionary reasons accounting for such complex shape and morphology are not uniquely determined yet: the silica structure is surely multifunctional, since it can act as virus barrier, selective-size membrane, protection against dangerous light wavelengths [16]–[18]. Recently, we found that centric frustule focuses light into a focal plane within the cytoplasm, which could explain the migration of chloroplasts within the cytoplasm under varying light conditions [19]–[22]. These unpredictable optical properties of diatoms frustule have driven our research in two directions: exploit the photonic features of these nanostructured components, from basic up to applications, and, on the other hand, correlate light response to frustule morphology and ultrastructure.

In this work, we document optical properties of a highly symmetrical and sculptured frustule belonging to the genus *Arachnoidiscus* by means of theoretical/numerical and experimental techniques, such as transmission measurements, digital holography, finite element method calculations, scanning electron microscopy.

## Materials and Methods

### Arachnoidiscus sp. material

Single valves of *Arachnoidiscus* sp. frustules have been obtained from the AM671 sample of the Hustedt collection. The sample dates from 1945 and was taken in Port Townsend, Washington, USA. The particular species under investigation is still classified as undetermined. General information about history and geographical distribution of the genus and frustule structure for the known species can be found in Ref. [23]. Despite its occurrence, mainly on seaweeds of tropical coasts around the Pacific [2], very little is known about the living cells belonging to this genus. In our case, the average diameter of the valves was 210 µm. Single drops of buffer suspension containing the cleaned valves have been deposited and dried onto a quartz slide for optical characterization and onto a silicon chip for Scanning Electron Microscopy (SEM) characterization.

### Scanning Electron Microscopy (SEM) measurements

Images of *Arachnoidiscus* sp. have been recorded after valves deposition on flat silicon wafers, by drop casting. SEM images were performed at 5 kV accelerating voltage and 30 µm wide aperture by a Field Emission Scanning Electron Microscope (Carl Zeiss NTS GmbH 1500 Raith FESEM). Both secondary emission and in-lens detectors have been used.

### Atomic Force Microscopy (AFM) measurements

AFM measurements have been performed by means of XEI-70 microscope from Park Systems. The instrument is provided with two independent, closed- loop XY and Z flexure scanners for sample and tip, respectively. Flat and linear XY scan of up to 100 µm×100 µm with low residual bow is provided. Out of plane motion is less than 2 nm over entire scan range. Z-scan is up to 25 µm by high force scanner.

### Numerical simulations

Numerical simulations performed by RSoft CAD - Photonics Suite software are based on Beam Propagation Method (BPM) corrected for Wide Angles. Starting from the Helmoltz equation for components of the electric field:
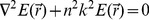
(1)with *E* electric field, *n* refractive index of silica and d *k = nk_0_* wavenumber (with *k*_0_ wavenumber in vacuum), we can write the field as:



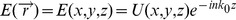
(2)We can thus separate the electric field into a slowly varying envelope factor *U(x,y,z)* and a rapid varying phase factor 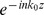. We are assuming that the considered wave propagates primarily along z (*paraxial approximation*), which is not true for a diatom valve where diffraction diverges light; we will see later how to overcome this limit. Furthermore, we are assuming that the profile along xy plane varies slowly and the amplitude varies slowly along z axis. Inserting 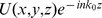 into equation (1) gives:
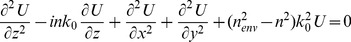
(3)with *n_env_* refractive index of the environment in which the diatom valve is immersed (air, water, or cytoplasm). Making use of the *Slowly Varying Envelope Approximation*:
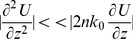
(4)we can get the basic BPM equation:



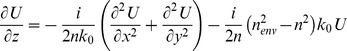
(5)Specifying *U(x,y,z)* at a plane *z = z_0_*, we can iterate *U* along the z-axis using finite differences for the x and y derivatives.

In order to derive a wide-angle (*non paraxial approximation*) BPM, we can consider the Helmoltz wave equation expressed in terms of the slowly varying fields (eq. 3) by neglecting the 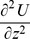 term. Denoting 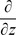 with *D*, and, thus,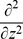 with *D^2^*, equation (3) can be now viewed as a quadratic equation to be solved for the differential operator *D*. This yields to the following formal solution for a first order equation in *z*:
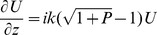
(6)with:



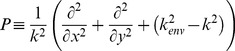
(7)Althought restricted to forward propagation of the field, the above equation is still exact in that no paraxiality approximation has been made. In order to evaluate the radical in equation (6), one approach would be to use a Taylor expansion. The first order of the expansion lead to standard, paraxial BPM while higher orders lead to more accurate representations. However expansion via Padé approximations is more accurate than Taylor expansion for the same order of terms [22]. This approach leads to the following wide-angle equation:
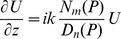
(8)where *N_m_* and *D_n_* are polynomials in the operator P, and *(m,n)* is the order of approximation. Our numerical capabilities allowed us to make use of the (1,1) Padé order, corresponding to *N_m_* = P/2 and *D_n_* = 1+P/4.

### Transmission measurements

**Figure 1:**
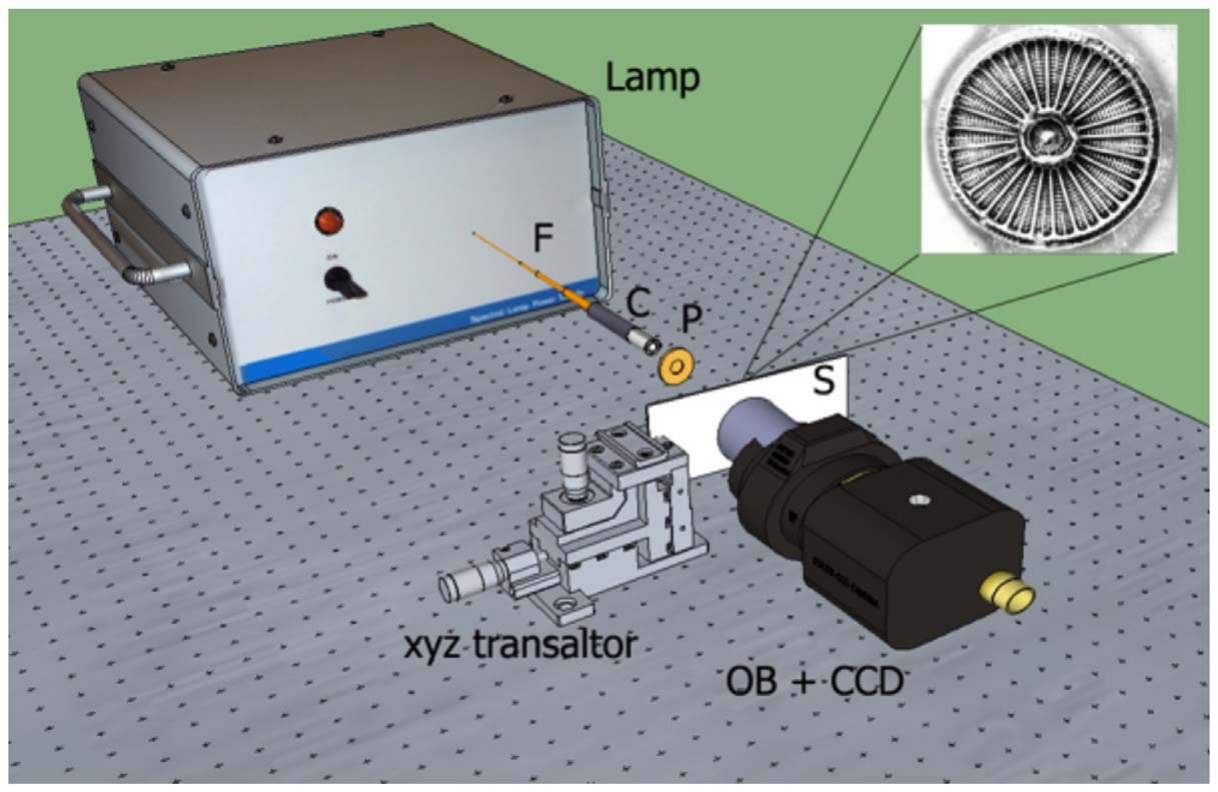
Experimental optical set-up. The source is given by a switchable, filtered deuterium/halogen lamp with fiber (F) output. The divergent radiation is partially collimated by collimator C. A metallic pinhole, 200 µm in diameter, spatially filters the incoming radiation. On quartz slide S sparse valves of *Arachnoidiscus* are deposited and selected by the xyz micro-stage translator. Finally, the transmitted light is collected by a microscope objective (OB) coupled with a CCD camera. All the optics and detectors are transparent and/or sensitive in UVB-VIS (see text).

Spatial distribution of light intensity transmitted by individual valves in different regions of the optical spectrum has been retrieved by means of the experimental set-up described in detail in Figure 1. The source of partially coherent radiation is given by a UV-VIS lamp (Hamamatsu, model L10290) provided with optical fiber output; this source includes a deuterium lamp (with emission in the spectral range 200–400 nm) and a tungsten halogen lamp (with emission in 400–1100 nm interval). The two lamps can be used independently or simultaneously, and, in general, the source is provided with a filter holder which can accommodate optical band-pass filters to select limited spectral regions for emission. We used filters at the following spectral windows: 280–315 nm (Asahi Spectra, UVB filter), 460 nm (Thorlabs, FL460-10, 10 nm FWHM), 532 nm (FL532-10, 10 nm FWHM), and 640 nm (Thorlabs, FB640-10, 10 nm FWHM). The radiation at the selected spectral window is emitted through a connected fiber (Hamamatsu, A7969 anti-solarization fiber) and collimated by a quartz collimator C (Lot Oriel, LLZ010), then spatially filtered by a metallic pinhole P (diameter: 200 µm) in order to produce a light beam with comparable dimensions respect to the analyzed valve. The valve is deposited onto a quartz slide, and selected by means of a micrometric xyz translational stage. The transmitted light is collected by a microscope objective (Zeiss, 50X Epiplan, NA 0.7 for visible measurements; Thorlabs, LMU-20X-UVB with AR coatings in 240–360 nm range for UVB measurements) connected with a UVB-VIS-NIR sensitive CCD camera (Hamamatsu, C8484-16C, QE 20–40% for 200–280 nm, 20–32% for 280–580 nm and below 20% between 580 and 1100 nm). The acquired images are analyzed and compared with light transmitted by a portion of the quartz slide without any valve. All the optics and the detectors are then transparent and/or sensitive in the UVB-VIS range.

### Digital holography measurements

**Figure 2:**
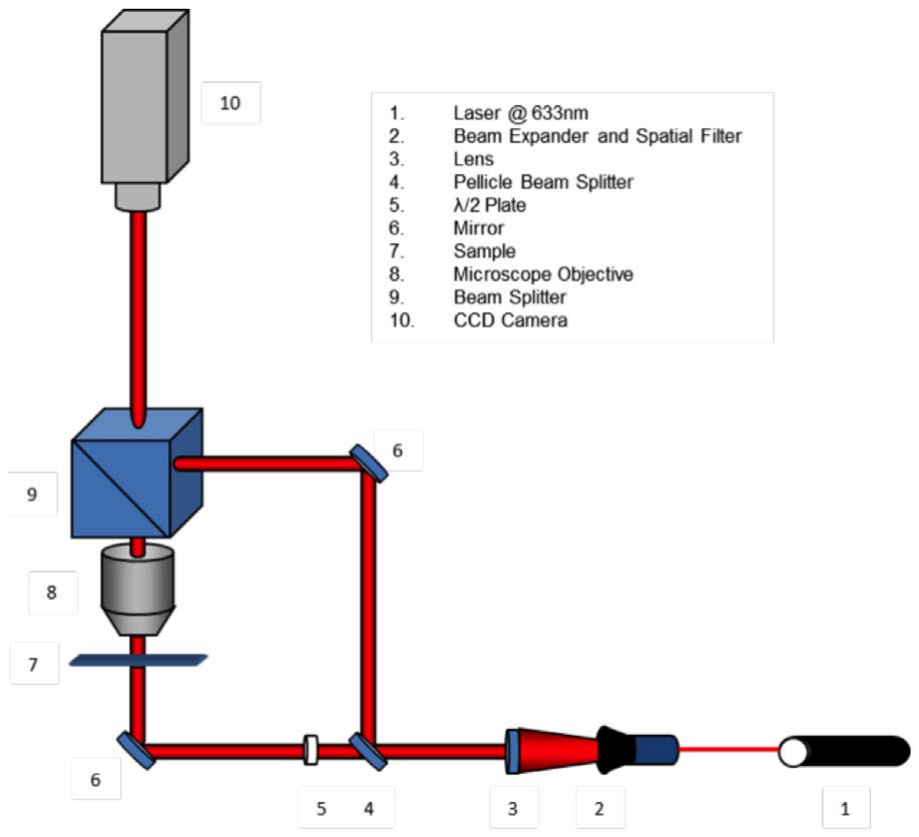
Experimental set-up for digital holographic characterization.

Digital holographic characterization has been realized with a Helium-Neon laser source (λ = 632.8 nm), with an output power of 30 mW in CW. The reference and object beams (see Figure 2 for details), obtained by a beam-splitter, passed through two beam expanders. A λ/2 wave plate was in the object beam in order to obtain an equal polarization direction for the two beams, improving the fringe contrast of the interference pattern. The object beam was collected by a microscope objective with a magnification and numerical aperture (NA) of 10X and NA = 0.25, respectively. The beams were recombined by a second beam-splitter and the resulting interference pattern has been collected onto the surface of a CCD camera (1392×1040 pixels array; each pixel had dimension Δx = Δy = 4.7 µm).

The image reconstruction procedure allows to retrieve a discrete version of the complex optical wavefront diffused by the object under test. The complex field of the object beam is reconstructed numerically from the frequency spectrum of the acquired hologram. In order to obtain the spatial separation of three diffraction terms without overlapping, a configuration with a small angle between the reference beam and the object beam (*off-axis configuration*) is adopted. Thus, the first diffraction order can be separated from the whole spatial frequency spectrum with a bandwidth filter and shifted to the origin of the plane, obtaining the spectrum of the object field (defined as 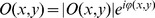, with 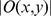 and 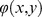 amplitude and phase, respectively, and x and y cartesian coordinates defining the plane of acquisition of the hologram), except for a constant [24]. Since the whole field is known, it is possible to reconstruct the optical wavefront at different distances from the plane of acquisition applying the Fourier formulation of the Fresnel-Kirchhoff diffraction formula [25]. An interesting approach for the analysis of the propagation problem by means of the operator algebra has been proposed by J. Shamir [26]. Fresnel diffraction is described by replacing the Fresnel-Kirchhoff integral, the lens transfer factor, and other operations by operators. The resulting operator algebra leads to the description of Fourier optics in a simple and compact way, bypassing the cumbersome integral calculus. By means of this approach the propagated field 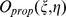 as a function of the initial field *O*(*x, y*) can be rewritten as
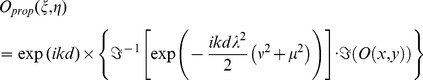
(9)being 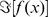 Fourier transform of the function *f(x)*,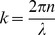 (with *n* refractive index of the medium), *ν* and *µ* spatial frequencies defined as 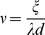 and 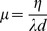, and *d* the reconstruction distance. For digital reconstruction Eq. (9) is implemented in a discrete form:
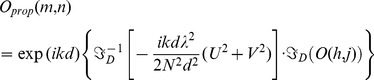
(10)where *N* is the number of pixels in both directions, *m*, *n*, *U*, *V*, *h* and *j* are integer numbers varying from 0 to *N*−1. The discretized Fourier transform is defined as



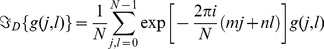
(11)Intensity and phase distributions can be retrieved starting from the propagated field according to the following relations:
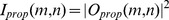
(12)




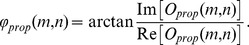
(13)


Since the phase distribution is obtained by a numerical evaluation of the *arctan* function, the values of the reconstructed phase are restricted in the interval [*−π*, *π*], i.e., the phase distribution is wrapped into this range. In order to resolve possible ambiguities arising from thickness differences greater than *λ*/2, phase-unwrapping methods have to be generally applied. The possibility offered by DH to manage the phase of the reconstructed image allows to remove and/or compensate the unwanted wavefront variations (such as optical aberrations, slide deformations etc.). In our experiments, a double exposure technique is used. The first exposure is made on the sample under investigation, while the second one is made on a flat reference surface in proximity of the object. Information about all the aberrations introduced by the optical components, including the defocusing due to the microscope objective, is incorporated into the second acquired hologram. In such a way, it is possible to compensate these aberrations by numerically manipulating the two holograms.

## Results

### Frustule Morphology Characterization

**Figure 3:**
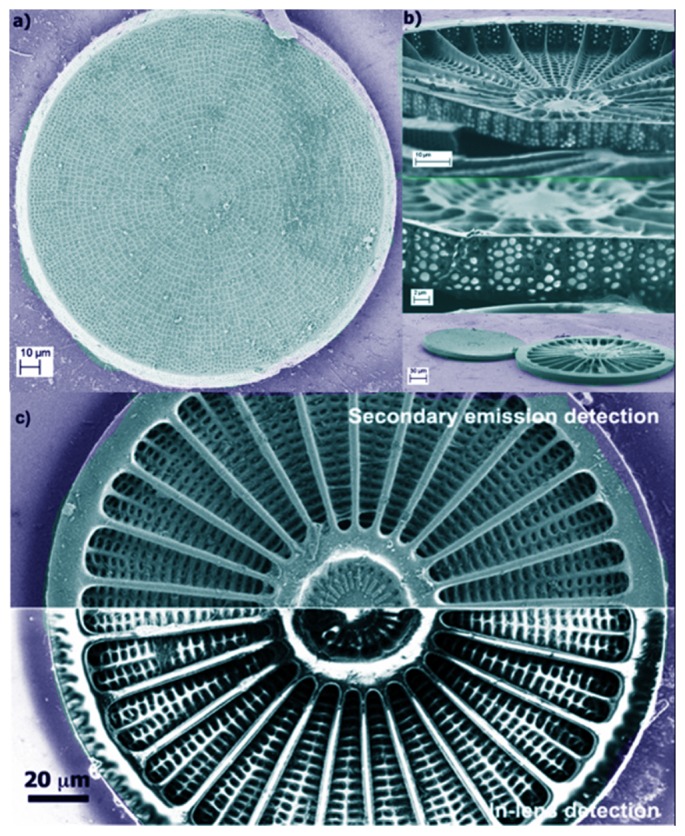
Details of a single valve of *Arachnoidiscus* genus obtained by a Field Emission Scanning Electron Microscope (FESEM). a): external plate; b): details of the internal plates, showing also the *costae* radiating from the central flange. c): Inner valve of *Arachnoidiscus* detected by two different SEM techniques: secondary emission detector (upper part of figure) shows the peculiar topology of the valve, whereas the in-lens detector (lower part of figure), by collecting the back scattered electrons, underlines the material composition of the valve and, consequently, different thicknesses of the same material. Scale bars: 40 µm (a), 20 µm (b, upper plate), 8 µm (b, middle plate), 90 µm (b, lower plate), 60 µm (c).

**Figure 4:**
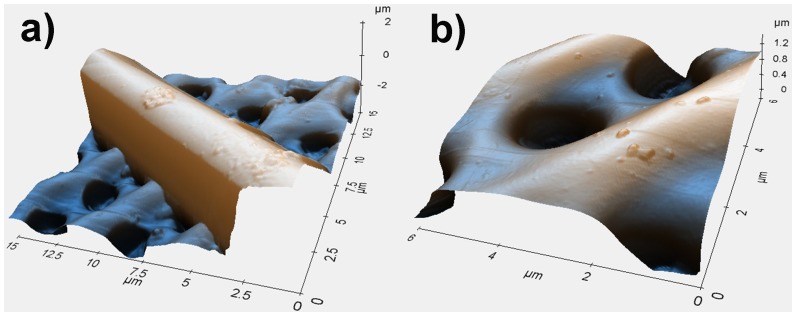
Details of a single *costa* (a) and single pores (b) of a valve obtained by means of Atomic Force Microscopy (AFM).

**Figure 5:**
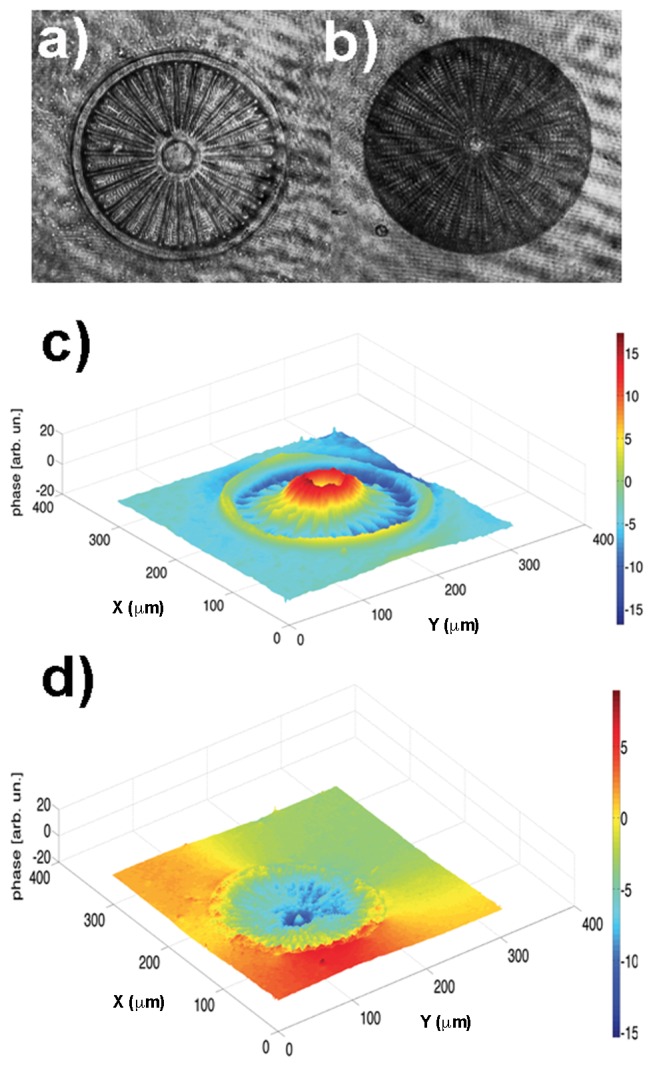
Holograms acquired by a CCD camera for *Arachnodiscus* diatom in upper (a) and lower (b) side of the valve and (c), (d) their three-dimensional phase reconstructions.

Figure 3 shows SEM images of a single valve of *Arachnoidiscus* sp. with some details at different magnifications. Frustules of *Arachnoidiscus* are heterovalvar, containing two different valves, and the whole frustule is Petri dish shaped. One valve is characterized by a planar central area (see Figure 3a) and the second one by a centre ringed with elongated radial slits (not shown). In general, the valve presents a clear ultrastructure characterized by progressively reduced porous features, according to position with respect to the plate, with dimensions ranging from micrometers to tens of nanometers. The internal side of the valve (see Figure 3c) hosts a system of *costae* radiating from a flange around a central ring. Figure 4 shows a further morphological characterization of a single *costa* and single pores of the valve obtained by means of Atomic Force Microscopy (AFM). It can be noticed how the *costa* is in relief with respect to the lying plane of the pores. Finally, Figure 5 shows the morphological characterization of a single valve by Digital Holography (see Methods). The thickness of the inner flange of the valve is clearly visible (see Figure 5c).

### Numerical simulations

**Figure 6:**
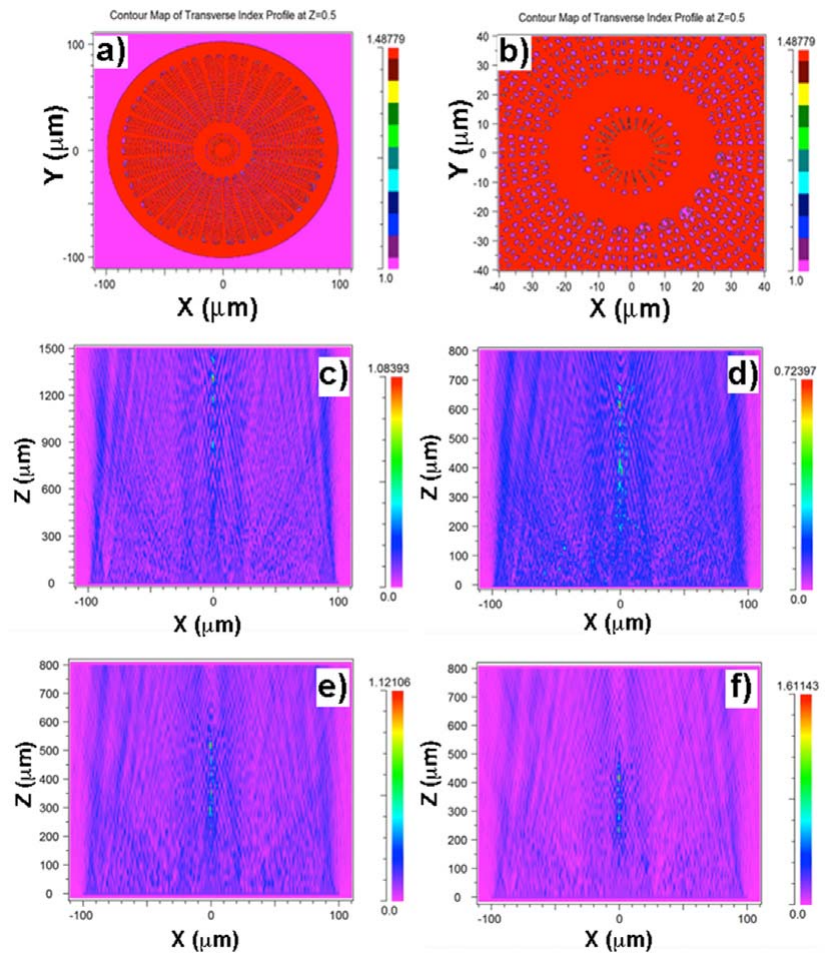
2D refractive index map of a single valve of *Arachnoidiscus* diatom (a) and relative detail (b) retrieved by a corresponding SEM image. The map has been successively extruded in order to obtain a simplified 3D model of the valve. Simulated, transmsitted power through a single diatom valve along the XZ plane at 300(**c**), 460 nm (**d**), 532 nm (**e**) and 640 nm (**f**). The radiation is propagated in air and the valve lies in XY plane for z = 0 (as long as its middle is concernerd). The diffraction contribution from valve edges, pores and elongated *costae* are clearly visible, interfering along the optical axis and giving rise to a train of hot spots. According to diffraction laws, lowering the wavelength increases the distance of the spot train from the valve plane. Please note the different scales.

Wide Angle Beam Propagation Method (WA-BPM) based on multi-steps Padé-wide angle technique [22] (implemented by RSoft Photonics, RSoft Design group, http://www.rsoftdesign,com), allowed us to perform simulation of propagation of light at different wavelengths through a simplified model of an *Arachnoidiscus* valve (see Figure 3) obtained by extruding a SEM image of its inner plate. Even with such a simplified model, interesting results can be predicted (see Figure 6): the diffraction contributions arising from the valve edges, central flange, radial *costae* and pores interfere along the optical axis giving rise to a train of hot spots, whose relative position respect to the diatom valve is a precise function of wavelength, according to diffraction laws. Indeed, having in mind the direct dependence of the divergence angle of diffracted light from wavelength, we can see how, at 300 nm (wavelength which is harmful for DNA of organisms), the spots take place quite far from the valve (starting around 900 µm from the valve plane). Increasing wavelength, the train of hotspots furtherly approach the valve: at 640 nm, where clorophylls have one of their maxima in absorption, the hot spots start to form around 200 µm. Mature frustules are characterized by multiple girdles [23], so in these stages the height of the frustule is of the same order of magnitude of the valve, i.e. in these conditions confined light lies inside the cell. Note, furthermore, that these simulations are performed in air, while in cytoplasm the train of spots for visible light form even closer to the valve [17], [18]. In case of UVB, the light confinement takes place far away from the volume occupied by the frustule.

### Transmission measurements of non-coeherent light through a single valve

**Figure 7:**
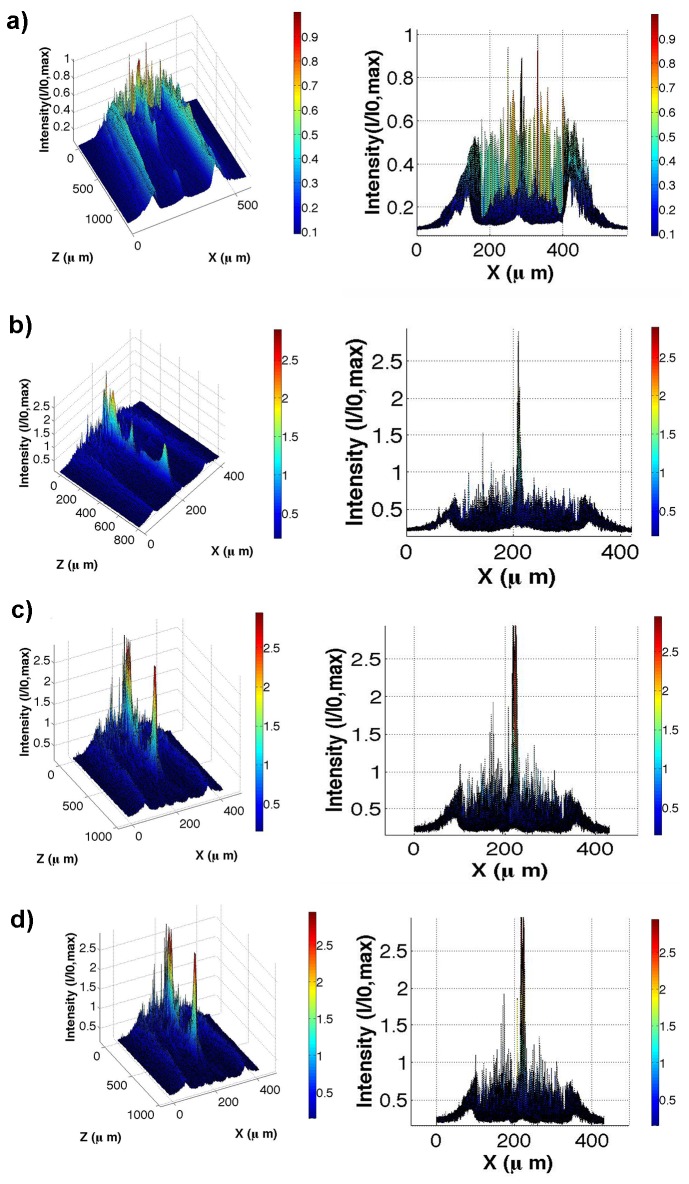
Transmitted intensity profiles, for different positions along the optiocal z axis and for a single diatom valve. (**a**): UVB; (**b**): blue; (**c**): green; (**d**): red. All the curves are normalized to the maximum of the incoming intensity profiles. On right side, a front view allows to better understand attenuation/amplification factors. Please note the different extensions along the z axis for the different wavelengths.

**Figure 8:**
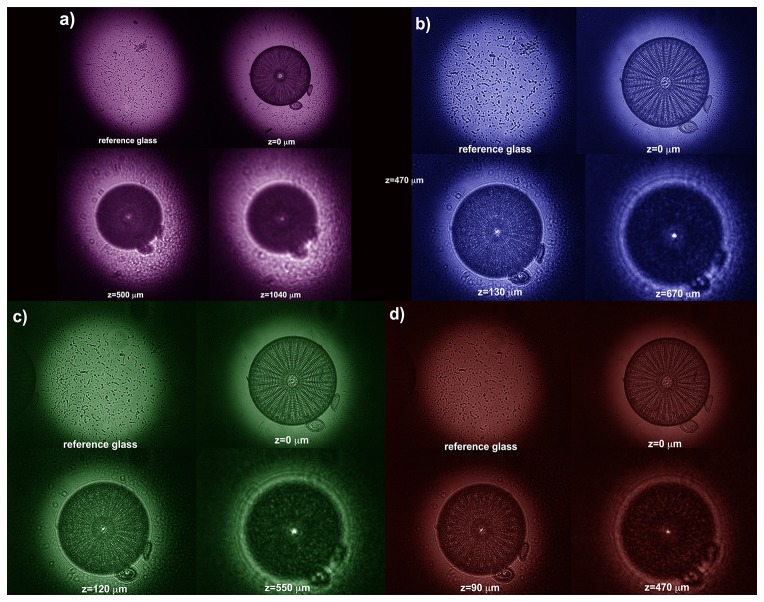
Transmitted intensity patterns in XY plane for UVB (a), blue (b), green (c) and red (d) illumination for the reference glass slide (left up side), for the valve on the focal plane of the collecting objective (z = 0 µm) and for the first two hot spots. Increasing wavelength, the focal spots get closer to the valve.

The acquired transmitted profiles at different spectral windows are reported in Figure 7, where intensity profiles along a diameter of the valve evaluated at different z positions along the optical axis are reported. We can notice, in accordance with the numerical simulation previously showed, that the main distance of the hot spots from the valve (z = 0 position along the optical axis) decreases while increasing the wavelength (see also Figure 8). Furthermore, the intensity in the hotspots, in case of visible light, is strongly amplified by means of constructive interference. In particular, for green and red light, even lowering at the minimum the integration time of the CCD camera and making use of high-density optical filters, the signal saturates. On the other hand, for UVB radiation, the spots intensity is quite low, comparable with incident radiation and the spots themselves are very distant from the valve. At this point it is interesting to distinguish between the main mechanisms which determine the attenuation of UVB radiation through a single valve of a diatom. First of all, for hydrated SiO_2_, the presence of water or OH absorption in the samples makes the determination of the intrinsic attenuation coefficient k values extremely difficult (if not impossible) in certain regions of the infrared and vacuum ultraviolet spectral regions [27]. A further role of hydration of diatom silica is UV-induced photoluminescence due to the presence of Si-OH and Si-H groups and non-bridging hole centers in nanostructured porous silica [28], with consequent emission of photosintetically active radiation (PAR) [10], [28]. On the other side, the presence of trace organic and inorganic materials embedded in diatom silica walls, which has been recently proved for *Stephanopyxis turris* by means of Raman and FTIR spectroscopy [29], can be identified as one of the possible origin of the UV absorption.

Besides absorption due to impurities incorporated in the hydrated silica matrix, also the geometry of the frustule is such that UV radiation is diffracted far away from the valve. It has to be noticed that, in order to avoid to detect visible photoluminescence induced by UVB excitation, a further bandpass filter has been inserted in the inlet of CCD camera, only for UVB measurements, in order to cut-off every possible visible contribution (see Methods section).

The study of the transmission of non-coherent light through a single valve of a diatom is of fundamental importance since, in their own environment, diatoms interact with non-coherent light par excellence, sunlight.

### Reconstruction of the transmitted optical field by Digital Holography

**Figure 9:**
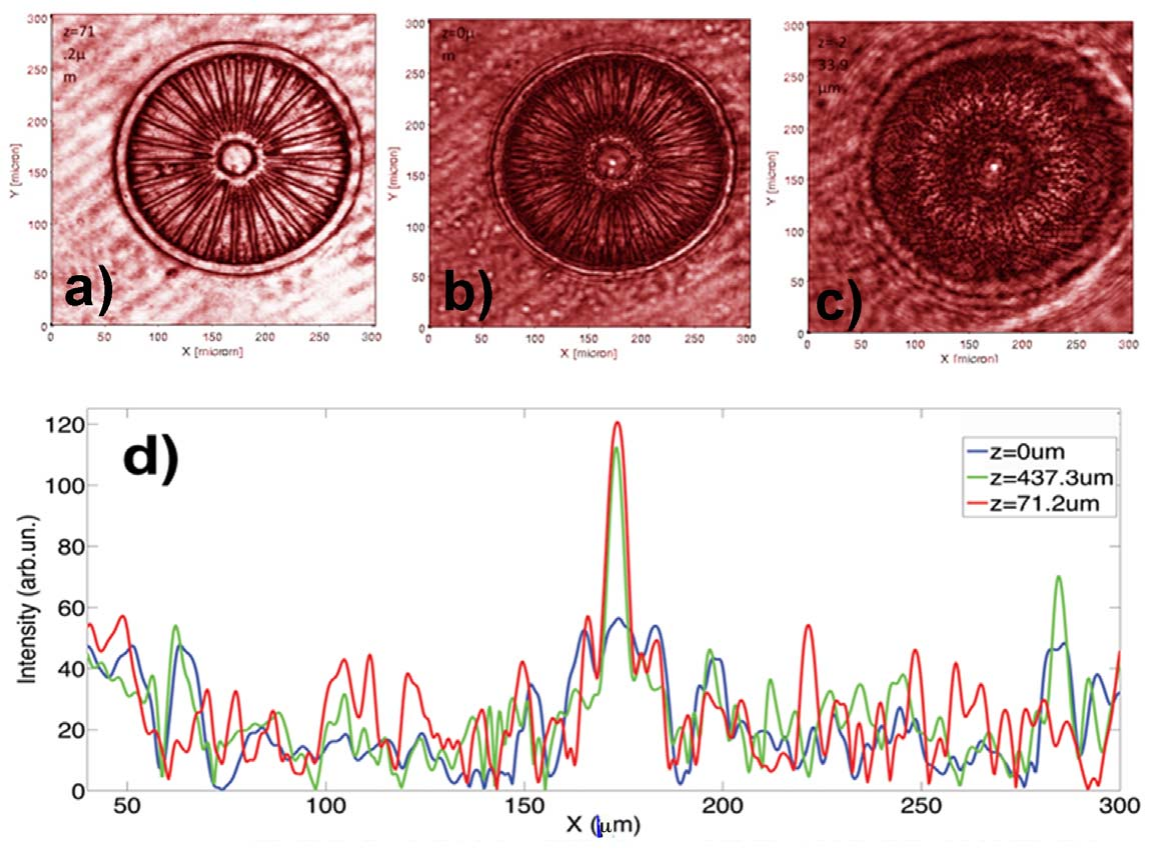
Intensity maps obtained by Digital Holography at λ = 632.8 nm at different distances along the optical axis: a) in the focus plane (*z* = 0 µm); b) and c) forward the focus plane (*z* = 71.2 µm and *z* = 437.3 µm). Light confinement occurs in a zone along the optical axis corresponding to the center of the diatom. d): intensity profiles obtained at different distances along the optical axis in the focus plane (*z* = 0 µm, blue line) and forward the focus plane (*z* = 71.2 µm, red line; *z* = 437.3 µm, green line).

The reconstructed intensity profile of the valve is reported in Figure 9 (a–c). Digital Holography (DH) allows the reconstruction of the transmitted intensity field along *z*-direction by means of a proper numerical algorithm (for analytical details see equation (11) in Methods). Only one hologram has been acquired and then the reconstructed field was propagated with a scanning pitch of ≈λ/20 along *z* direction, thus giving a more resolved characterization of the *z*-propagation with respect to typical measurements which require acquisition of series of images at different positions along the optical axis by means of a microscope objective (see Ref. [18]–[20] and previously shown measurements). By using the aforementioned algorithm, the reconstructed optical wave field has been propagated at different distances. In Fig. 9 reconstructions of the light transmitted by the diatom valve in air at two different distances are reported: *z* = 71.2 µm (b), *z* = 437.3 µm (c). Light confinement occurs, along *z* direction, in correspondence of the center of the diatom and eventually also a light ring appears, probably due to the diffraction from valve edges and *costae*. The intensity profiles at *y* = 163 µm (along the diameter of the valve), at all the three distances considered, are showed in Fig. 9d. The Full Width at Half Maximum (FWHM) of the spot is 9.2 µm at *z* = 71.2 µm, and 10.1 µm at *z* = 437.3 µm. In these measurements, the incident beam is expanded up to 1 cm diameter, collimated and then directed on the frustule: the coherent light is thus squeezed by a factor greater than 1000.

## Discussion

In this study, we made use of several numerical and experimental techniques in order to fully characterize the optical properties of a single valve of the centric diatom *Arachnoidiscus*, which in nature lives adherent to seaweeds (epiphyton), mainly along the Pacific coasts [2]. Seaweed beds can experience low access to sunlight due to mutual shading of suspended and attached algae absorbing and processing light for their photosynthetic reactions, so it is likely that the studied diatom, as other centrics [17]–[19], had developed, in the course of their evolutionary history, fine optical mechanisms in order to optimize PAR collection and to minimize exposure to noxious UV radiation and consequently photodamage. Indeed, as long as transmission measurements are considered, the investigated spectral regions are of great importance in diatom biology since chlorophyll a, the primary pigment responsible of the photosynthetic process, mainly absorb in blue and red portions of visible spectral range while carotenoids, also involved in photosynthesis, mainly absorb in green [30]. The study of UVB-diatom valve interaction is, on the other side, very important in order to assess in which terms silica nanopatterned valves are able to protect living organisms from DNA harmful radiation. Evolution seems to have shaped frustules in order to maximize visible light exploitation and to reduce at most the damage induced by UVB irradiation; it is thus clear that the evolutionary advantage of frustules relies not only on mechanical properties, but also on the possibility to manipulate light in a diversified way according to wavelength.

However, this study is not only of great importance in order to deeply understand the role of frustules in diatoms evolution, but also to introduce possible technological applications of these properties. For example, the ability to confine light in a train of hotspots whose position along the optical axis changes varying wavelength could lead to the exploitation of single diatom valves as micro-monocromator elements in MOEMS.

## Conclusions

Light manipulation by means of single valves of *Arachnoidiscus* sp. has been characterized by means of different optical techniques. Incident light is confined in a train of discrete spots, exploiting diffraction laws and following different behaviors in different spectral regions of em spectrum. This study opens a new breakthrough in the understanding of the evolutionary advantage given by the silica frustules to diatoms, whose success in colonizing almost all habitats is highly documented; on the other side possible applications in optical microsystems of diatom frustules and frustule-inspired devices as active photonic elements can be envisaged.
